# Hepcidin mediates hypoferremia and reduces the growth potential of bacteria in the immediate post-natal period in human neonates

**DOI:** 10.1038/s41598-019-52908-w

**Published:** 2019-11-12

**Authors:** Sarah Prentice, Amadou T. Jallow, Edrissa Sinjanka, Momodou W. Jallow, Ebrima A. Sise, Noah J. Kessler, Rita Wegmuller, Carla Cerami, Andrew M. Prentice

**Affiliations:** 1 Clinical Research Department, London School of Hygiene and Tropical Medicine, Keppel Street, London, WC1E 7HT United Kingdom; 2 Nutrition Theme, MRC Unit The Gambia at The London School of Hygiene and Tropical Medicine, Fajara, Gambia; 3 GroundWork, Flaesch, Switzerland

**Keywords:** Translational research, Paediatric research

## Abstract

Septicemia is a leading cause of death among neonates in low-income settings, a situation that is deteriorating due to high levels of antimicrobial resistance. Novel interventions are urgently needed. Iron stimulates the growth of most bacteria and hypoferremia induced by the acute phase response is a key element of innate immunity. Cord blood, which has high levels of hemoglobin, iron and transferrin saturation, has hitherto been used as a proxy for the iron status of neonates. We investigated hepcidin-mediated redistribution of iron in the immediate post-natal period and tested the effect of the observed hypoferremia on the growth of pathogens frequently associated with neonatal sepsis. Healthy, vaginally delivered neonates were enrolled in a cohort study at a single center in rural Gambia (N = 120). Cord blood and two further blood samples up to 96 hours of age were analyzed for markers of iron metabolism. Samples pooled by transferrin saturation were used to conduct *ex-vivo* growth assays with *Staphylococcus aureus, Streptococcus agalactiae, Escherichia coli* and *Klebsiella pneumonia*. A profound reduction in transferrin saturation occurred within the first 12 h of life, from high mean levels in cord blood (47.6% (95% CI 43.7–51.5%)) to levels at the lower end of the normal reference range by 24 h of age (24.4% (21.2–27.6%)). These levels remained suppressed to 48 h of age with some recovery by 96 h. Reductions in serum iron were associated with high hepcidin and IL-6 levels. *Ex-vivo* growth of all sentinel pathogens was strongly associated with serum transferrin saturation. These results suggest the possibility that the hypoferremia could be augmented (e.g. by mini-hepcidins) as a novel therapeutic option that would not be vulnerable to antimicrobial resistance. Trial registration: The original trial in which this study was nested is registered at ISRCTN, number 93854442.

## Introduction

It has been estimated that 2.9 million neonates die each year from largely preventable causes; 600,000 of these from neonatal infections^1^. With the rapid spread of antimicrobial resistance (AMR), these statistics are likely to worsen^2^. AMR frequently contributes to neonatal septicemia in low-income countries (eg for *Klebsiella* spp, *E. coli* and *S. aureus*), and is almost certainly rising^3^. Poor susceptibility to almost all commonly-used antibiotics has been reported for *Klebsiella* species and *S. aureus* in neonatal settings^4^. AMR is especially devastating for neonatal care units because babies succumb rapidly and often before it is possible to screen for AMR or try alternative antibiotics. Against this background, there is a pressing need to better understand why neonates are so susceptible to blood-borne infections and to develop adjunctive therapies that could aid their protection perhaps by augmenting first-line innate responses.

The growth and virulence of most human pathogens is contingent on their ability to assimilate iron from their human host. High host iron states can lead to increased susceptibility to many infectious diseases^5^. As a result, systemic iron homeostasis in humans is tightly controlled; a process mediated primarily by hepcidin^5^, and possibly also by hepcidin-independent pathways in response to infectious threat^6^. In the acute phase response hepcidin is rapidly up-regulated by inflammatory cytokines (primarily IL-6). Raised hepcidin levels cause internalization of the transmembrane protein ferroportin in enterocytes and macrophages. This reduces serum iron by blocking enteric absorption of dietary iron and sequestering iron in macrophages^5^. The reduction in serum iron with inflammation is believed to be an evolutionary mechanism designed to withhold iron from microbes and thus limit their growth and virulence and is viewed as a key component of nutrient-mediated innate immunity. The effectiveness of this process has now been clearly demonstrated in mouse models^7–9^.

Neonates are born with high levels of fetal hemoglobin, ferritin, serum iron and transferrin saturation (TSAT) as evidenced by cord blood levels^10^. The physiological challenge of dealing with high heme levels at birth is illustrated by the fact that around half of all neonates show transient jaundice^11^. We therefore hypothesized that these elevated iron levels and fluxes might contribute to the high susceptibility of neonates to septicemia, especially preterm and low birth-weight babies, and may partially explain the characteristic spectrum of causal organisms. Here we report that healthy vaginally-delivered African babies display a very rapid post-natal hypoferremia that is correlated with changes in IL6 and hepcidin. We suggest that this represents an evolved protective mechanism that could potentially be augmented to provide a broad-spectrum innate protection against neonatal septicemia.

## Results

### Neonatal characteristics

Baseline demographics for the 120 study participants are shown in Table 1. Children in this cohort were healthy term infants, with median anthropometric measurements falling between the 25^th^ and 50^th^ centile on the WHO growth charts for gestational age. Nearly all (97.5%) of mothers received iron and folic acid antenatally, as per WHO guidelines. Six infants in the cohort became unwell during the study period (five with suspected sepsis, one with suspected meconium aspiration) and were excluded from analysis.

**Table 1:** Baseline Characteristics of the study population.

Characteristic	Median (IQR)
Gestational Age (weeks)	38 (37–40)
Birth weight (g)	3085 (2858–3325)
Head circumference (cm)	34 (33–35)
Length (cm)	51 (49–52)
Maternal parity	3 (1–6)
Percentage male (%)	49%
Percentage of mothers on antenatal iron/folic acid supplementation at recruitment	97.5%
Age at post-natal blood sampling (hours)	
<24 hour sample (S1)	6 (2–11)
24–48 hour sample (S2)	29 (26–34)
72–96 hour sample (S3)	77 (74–82)

Data are presented as median followed by the Interquartile Range in parenthesis or as a percentage of the total population studied.

### Alterations to iron metabolism in the acute post-natal period

**Figure 1:**
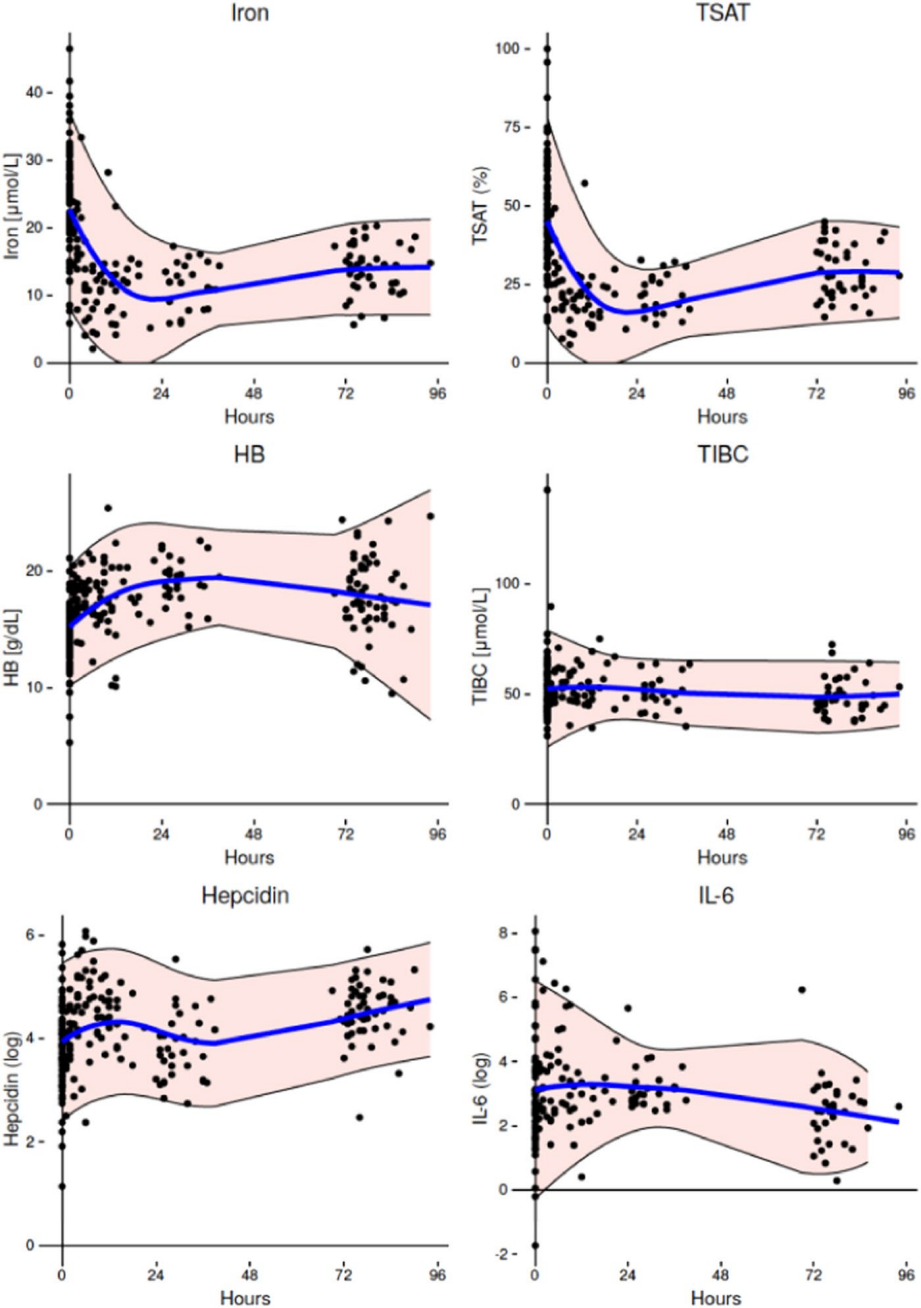
Changes to iron markers during the first 96 hours of life. Blood was drawn from either the umbilical cord at birth or from the dorsum of the hand at the indicated times post-natal. Dots represent individual measurements. The blue line is a Loess fit curve with 95% Confidence Intervals shaded in pink.

Iron metabolism markers in the first 96 h of life are shown in Table 2 and Fig. 1. Mean TSAT was high in cord blood (47.6%, 95% confidence interval (CI) 43.7–51.5%) with levels higher than the reported reference range for older children. TSAT levels had halved by 12 h post-partum (24.4%, CI 21.2–27.6%) and remained low until 72–96 h when levels began to rise again (30.9%, CI 26.9–34.8%). TSAT alterations were largely driven by alterations in serum iron rather than by changes to the chaperone protein transferrin, as total iron binding capacity (TIBC) remained relatively constant, though showing a slight fall by 72–96 h of age. Geometric mean hepcidin levels in cord blood (43.8 ng/ml, CI 36.8–52.3 ng/ml) were within the expected reference range for healthy older children^12–14^, and had almost doubled by the first post-natal blood draw at a median time of 6 h post-partum (79.4 ng/ml, CI 68.1–92.4). This was followed by a decline at the subsequent sampling point at 24–48 h (45 ng/ml, CI 36.5–57.8) and a rise again by 72–96 h (87.1 ng/ml, CI 73.8–102.7). Geometric mean IL-6 levels decreased by nearly half between birth and 72–96 h. Cord blood hemoglobin levels (14.4 g/dl, CI 13.8–14.9 g/dl) were within previously reported reference ranges^15^. Levels then rose until 24–48 h of age (19.2 g/dl, CI 18.3–20.0 g/dl) and began to fall subsequently (17.9 g/dl, CI 17.0–18.7 g/dl) at 72–96 h of age as expected for this age group.

**Table 2:** Markers of iron metabolism by post-natal age.

	Cord blood n > 81***	Age < 24 hours (S1) n = 53	Age 24–48 hours (S2) n = 21	Age 72–96 hours (S3) n = 33
TSAT* (%)	47.6 (43.7–51.5)	24.4 (21.2–27.6)	21.8 (18.8–24.7)	30.9 (26.9–34.8)
Iron* (μmol/L)	24.7 (22.5–26.9)	13.6 (12.0–15.2)	11.6 (10.1–13.1)	14.5 (13.1–16.0)
TIBC* (μmol/L)	52.2 (49.0–55.4)	54.0 (51.4–56.6)	51.0 (47.3–54.7)	47.9 (45.3–50.4)
Hepcidin (ng/ml)**	43.8 (36.8–52.3)	79.4 (68.1–92.4)	45.9 (36.5–57.8)	87.1 (73.8–102.7)
IL-6 (pg/ml)**	23.7 (14.7–38.1)	26.9 (18.9–38.2)	24.4 (18.0–33.0)	10.7 (7.3–15.6)
Hb (g/dl)*	14.4 (13.8–14.9)	17.6 (17.1–18.2)	19.2 (18.3–20.0)	17.9 (17.0–18.7)

Data are presented as: mean and 95% Confidence Interval (*), geometric mean and 95% Confidence Interval (**). *** Indicates that the number of available results differs for each marker due to limitations in blood sample volume.

### Likely effectors of changes in iron metabolism in the acute post-natal period

Pearson pairwise correlation coefficients between the iron measurements (serum iron, TIBC, TSAT and Hb) and the putative regulators of iron (IL-6 and hepcidin) are shown in Supplementary Table 1. We focus the discussion here on the possible mediators of the acute post-natal hypoferremia. Day 1 hepcidin and IL-6 values were correlated with their respective cord levels ( + 0.66; p < 0.001 and + 0.37; p < 0.05 respectively) and Day 1 hepcidin was correlated with Day 1 IL-6 ( + 0.38; p < 0.01). Day 1 TSAT was correlated with cord TSAT ( + 0.54; p < 0.001) and there were similar correlations between cord and Day 1 serum iron ( + 0.55; p < 0.001) and TIBC ( + 0.64; p < 0.001). Day 1 TSAT was inversely correlated with Day 1 hepcidin (−0.47; p < 0.001) and IL-6 (−0.40; p < 0.05) and similarly for serum iron which was the major determinant of TSAT. At the later sampling points TSAT levels were not significantly associated with hepcidin but showed a strong inverse association with IL-6 in the 72–96 h interval (−0.70; p < 0.001). Hemoglobin levels were strongly correlated across time within babies but did not appear to influence any of the iron markers, hepcidin or IL-6.

### *Ex vivo* assays of growth of sentinel organisms

**Figure 2:**
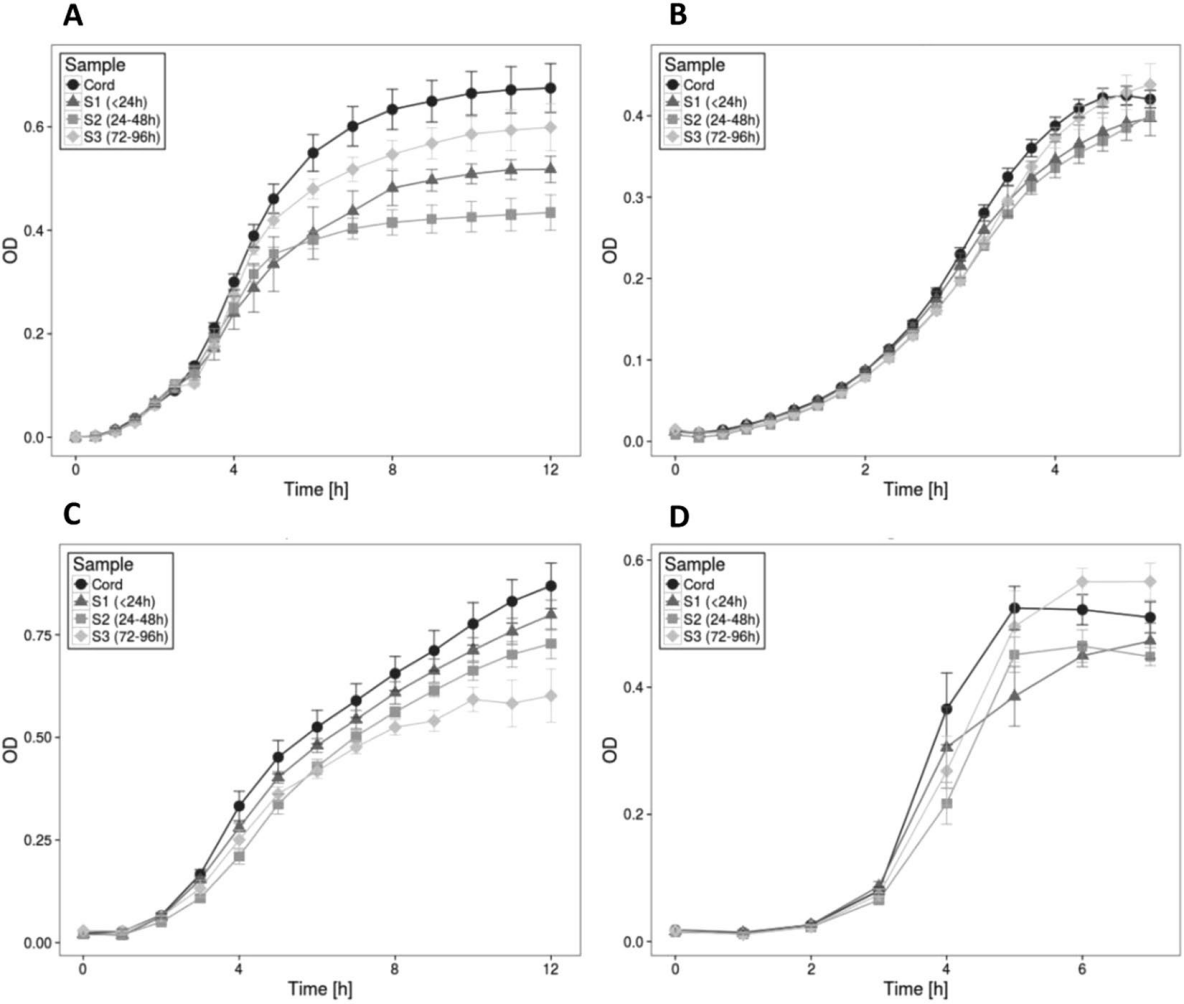
*Ex-vivo* bacterial growth assays. *Ex-vivo* bacterial growth assays Growth of *E.coli* (**A**), *K. pneumoniae* (**B**), *S. aureus* (**C**) and *S. galactaie* (**D**) in subject serum drawn from the umbilical cord (cord) or from the dorsum of the hand at the following time points after birth, S1 (<24 hours), S2 (24–48 hours) and S3 (72–96 hours). Dots represent the mean at each time point, error bars represent the SD. OD = Optical Density. Growth, was different between cord and S1/2/3 samples for all organisms (p < 0.01), with the exception of the comparison between cord and S3 samples for *K. pneumoniae* and *S. galactai*

The *ex vivo* growth patterns of standard lab strains of *Escherichia coli*, *Staphylococcus aureus*, *Klebsiella pneumoniae* and *Streptococcus agalactiae* were assayed in iron-free media supplemented with cord and neonatal serum pooled according to time and TSAT (Fig. 2). Post-natal sera clearly supported lower growth levels of all organisms and this was especially true of the Day 1 sera. The effect was least pronounced for *S. aureus*. Repeated measures ANOVA including incubation time and cord/neonatal sampling time confirmed that growth rates of all four organisms were significantly associated with TSAT (p < 0.001).

## Discussion

We demonstrate that normal healthy term newborns display a rapid and profound suppression of serum iron and TSAT within the first 6–12 h post-partum. This reduction in extracellular iron persisted until 2–3d of age, with a slight increase subsequently. The correlation of suppressed iron and TSAT levels with raised hepcidin levels, particularly in the first 24 h of life, suggests that hepcidin regulation of iron homeostasis is intact in the human neonate and that this is likely to be the key mediator of the hypoferremia through redistribution of iron to macrophages. Similar correlations with raised IL-6 levels (10–20 fold higher than normal adult levels), suggest that inflammatory stimulation of hepcidin also occurs in early life, and that the inflammatory conditions induced by the birth process may be at least partly driving the hypoferremia of early post-natal life. However, correlations between iron markers and IL-6 were weak, and have not been observed in previous studies looking at cord blood markers of iron metabolism^16^. This could suggest that other unmeasured inflammatory mediators, such as IL-22^17^ may also be up-regulating hepcidin in response to the birth process.

Two previous reports have similarly reported low iron levels in post-natal blood draws^18,19^. The data from Szabo *et al*. were based on 10 infants who were sampled due to clinical indications (jaundice or infection) at a mean post-natal age of 48 ± 4 h^19^. Serum iron decreased from 23.2umol/l in cord to 7.2umol/l post-nataly (arithmetic means). The data from Sturgeon based on 72 infants sampled by 12 h post-partum showed a decrease from 193ug/100cc (34.5umol/L) to 46ug/100cc (8.2umol/L) (arithmetic means). These compare favorably with our values of 24.7 vs 13.6umol/L (geometric means) by 6 h (IQR 2–11 h)^18^. Thus, we clearly demonstrated that normal neonates elicit a rapid and profound (2–4 fold) decrease in extracellular iron in the early post-natal period that is likely to be hepcidin mediated. Since newborns have a negligible iron intake from colostrum the hypoferremia must be achieved by redistribution of iron; presumably in macrophages where iron egress is blocked by hepcidin’s inactivation of the transmembrane iron exporter, ferroportin.

A previous study linked a fall in serum iron with an increase in the anti-oxidant capacity of post-natal serum, suggesting that this may protect new-born infants against free-radical damage during the transition from fetal to post-natal life^19^. In the present study, we hypothesized that the hypoferremia might be a protective mechanism to withhold iron from bacteria and other human pathogens. Early post-natal life is characterized by massive colonization of the skin and gastrointestinal tract with a variety of commensal organisms^20^. A reduction in the availability of serum iron may be an evolved innate mechanism to help prevent these organisms from overwhelming the immature adaptive immune responses of neonates. To test this, we devised micro -culture methods based on lab isolates of four organisms that frequently cause neonatal sepsis in sub-Saharan Africa. The growth rates of *E. coli, S. aureus, S. pneumoniae* and *S. agalactiae* were substantially lower in neonatal serum than in cord serum and for each organism growth rates were significantly associated with TSAT. *S. aureus*, which favors heme iron as a source^21^ was least responsive though still clearly influenced by TSAT. *E. coli* was most responsive which is consistent with the findings of the infamous studies of Polynesian neonates given intramuscular iron, where the intervention caused an increase in neonatal septicemia and a major shift towards *E. coli* as the most frequently identified causal organism^22^. Our *ex vivo* assays need to be interpreted with caution and will certainly not replicate conditions *in vivo*, but have been validated by titrating with differing concentrations of exogenous iron and are coherent with the known dependence of bacterial growth on iron supply.

The wider applicability of these findings may be limited because the study population was restricted to vaginally delivered, healthy neonates above 2000g from one area of West Africa. Nearly all (97.5%) of the infants were born to mothers receiving antenatal iron and folic acid supplementation, which may have altered levels of iron markers at birth. TSAT levels in our study were lower than those reported in a recent systematic review of cord blood iron markers (weighted mean TSAT 61.2%), although fell within the reported 2.5^th^-97.5^th^ centile range^10^. Cord blood hepcidin in our study was also lower than has been previously reported^10,16,23,24^, although the lack of a standardized immunoassay for hepcidin detection makes comparing levels between studies difficult. However, these results could suggest that despite almost universal iron supplementation, our study infants’ iron stores remained relatively lower at term than in other populations. This may indicate low adherence rates to supplementation or might reflect physiological differences in this population, for instance, reduced gut absorption of elemental iron (possibly due to chronic inflammation). It would, therefore, be interesting to see whether neonatal hypoferremia is even more exaggerated in different, iron replete, settings as hinted by the previous studies by Szabo^19^ and Sturgeon^18^. A recent prospective study characterizing hepcidin levels in cord blood also showed lower levels in premature infants, those born small-for-gestational-age and those delivered by elective caesarean section^16^. We have now initiated a study to test whether a blunting of the physiological hypoferremia of early neonatal life occurs in these situations, putatively increasing the potential for iron-induced free-radical damage and bacterial pathogenicity from low virulence organisms, such as is noted particularly in premature infant populations^25^.

Low plasma iron is bacteriostatic rather than bacteriocidal, but nonetheless could tilt the balance towards host survival by slowing the multiplication of pathogens that might otherwise overwhelm the immature adaptive defenses of newborns. If it were possible to artificially augment such responses this could form the basis of a novel intervention. Small molecule orally-administered mini-hepcidins are currently under development and first-in-human testing as hepcidin agonists^26^. These molecules would not affect the neonate’s longer-term iron status because they would only cause a transient redistribution of iron away from the circulation where it is most available to pathogens. Although it presently remains a distant prospect, hepcidin analogues might prove to be useful adjuvants in the face of the rapidly growing levels of antimicrobial resistance.

We conclude that healthy term neonates undergo a rapid and profound reduction in serum iron levels during the first 12 hours of life, at least partly mediated by the hormone hepcidin. This hypoferremia is likely to produce protection against common bacterial pathogens and may be an evolved innate immune strategy to protect the infant during the first few days of microbial colonization. Identification of situations where this physiological hypoferremia is blunted should be a research goal. Mechanisms to enhance this hypoferremia, such as hepcidin agonists, represent an exciting novel therapeutic target that would not be susceptible to the threat of antimicrobial resistance.

## Methods

### Participants and study procedures

Blood samples for this study were collected during a trial investigating the impact of different vaccination strategies at birth on the iron status of neonates. A detailed description of the study methods can be found elsewhere^27^. In summary, 120 healthy Gambian neonates were recruited on the first day of life and randomly allocated to receive either 1) routine immunizations at birth (Bacillus Calmette Guerin (BCG), Hepatitis B and Oral Polio Vaccine (OPV)) 2) Hepatitis B and OPV at birth, BCG vaccination delayed to after study completion (>72 h of age) or 3) all immunizations delayed until after study completion (BCG, Hepatitis B and OPV at >72 h of age). All infants had a placental cord blood sample, a neonatal blood sample taken within 24 h of birth (S1) and were then randomly assigned to have one further blood sample taken at either 24–48 (S2) or 72–96 (S3) hours of age. As none of the different vaccination strategies had a significant impact on neonatal iron metabolism^27^, the results from all groups were combined in this study to investigate the physiological changes in iron metabolism within the first 4 post-natal days.

Full informed consent for infant involvement in the study was obtained from pregnant mothers antenatally, and eligible infants were enrolled on the day of birth. Any healthy infant born to a consenting mother within the West Kiang region of The Gambia was eligible for inclusion, providing that they were not already enrolled in another research study. No gestational age limit was set, however, infants weighing <2000 g (more than 2 standard deviations from the average Gambian birth-weight) were excluded (one exclusion). Other criteria for infant exclusion were; severe birth complications (six exclusions), major congenital malformations (no exclusions), unwell at birth (two exclusions), mother with known HIV or TB (no exclusions), and infants with a known case of active TB within the same compound of residence (no exclusions). Most mothers received supplementary iron and folic acid as part of their routine antenatal care, as per WHO guidelines.

### Ethical approvals

The study was approved by The Gambia Government/MRC Joint Ethics Committee (SCC1325) and the London School of Hygiene and Tropical Medicine ethics committee (012–045). The study was conducted according to the principles of the Declaration of Helsinki.

## Laboratory Methods

### Blood collection and iron marker analysis

Whole blood was drawn from the umbilical vein at birth or from the dorsum of the hand at the indicated time points after birth, into Becton Dickson microtainer SST II Advanced collection tubes. Haematology values were measured on fresh whole blood drawn into EDTA microtainers (Becton Dickson, Oxford, UK) using a Medonic M-series haematology analyzer (Boule Diagnostics Int AB, Stockholm, Sweden). Iron markers were analyzed using plasma collected into lithium-heparin anti-coagulant using the automated Cobas Integra 400 plus (Roche Diagnostics, Basel, Switzerland). Plasma hepcidin and IL6 were measured in duplicate by ELISA, Bachem-25, USA and BD OptEIA, Oxford, UK respectively, as per manufacturers’ instructions as previously described^27^.

Due to low volume of residual blood, bacterial growth assays were performed on plasma samples that were pooled according to time of collection (Cord, S1 (6–24 h after birth), S2 (25–48 h after birth), and S3 (72–96 h after birth)) and then according to TSAT. The following sample pools were made and run in triplicate through the bacterial growth assays: Cord 70–100% (n = 6, pools = 4); Cord 60–69% (n = 12, pools = 6); Cord 50–59% (n = 15, pools = 5); Cord 40–49% (n = 20, pools = 9); Cord 30–39% (n = 14, pools = 2); Cord 20–29% (n = 10, pools = 3); Cord 10–29% (n = 4, pools = 1); S1 30–60% (n = 12, pools = 2); S1 20–30% (n = 26, pools = 2); S1 0–20% (n = 30, pools = 2); S2 20–30% (n = 12, pools = 2); S2 10–20% (n = 9, pools = 2); S3 30–40% (n = 15, pools = 2); S3 20–30% (n = 21, pools = 3); and S3 0–20% (n = 13, pools = 2).

### Bacterial growth assays

*Staphylococcus aureus* (strain NCTC8325), *Escherichia coli* (strain *Crooks*, ATCC8739), *Streptococcus agalactiae* Lehmann and Neumann (ATCC 13813, Lancefield’s group B) and *Klebsiella pneumoniae* (ATCC13883, strain NCTC96633) were grown overnight for 18 h at 37 °C in 5mls iron-free minimal growth media, Iscove’s Modified Dulbecco’s Medium (IMDM, Invitrogen) with continuous shaking (250 rpm). All growth assays were run in triplicate in IMDM containing 50% heat-inactivated human neonatal serum. Bacterial growth was monitored by measuring the optical density at 620 (OD_620_) hourly for 12 h using a Multiscan FC ELISA plate reader (Thermo Scientific).

### Statistical analysis

Statistical analysis and preparation of graphs was conducted using STATA v14.1 (Stat-Corp LP, College Station, TX, USA), DataDesk version 7.0.2 (Data Description Inc), GraphPad Prism (GraphPad Software INC, CA 92037, USA) and R (R: A Language and Environment for Statistical Computing, R Foundation for Statistical Computing, 2016, https://www.R-project.org).

Non-normally distributed markers (hepcidin and IL-6) were log-transformed prior to any analysis. Bacterial growth rates were compared using repeated measures ANOVA with pooled sample (discrete variable), cord/neonatal sampling period (discrete variable) and growth rate incubation time (continuous variable) as independent variables. Pearson product-moment correlation was used to obtain pair-wise correlations between iron markers. Graphs of changes in variables overtime were generated using local polynomial regression fitting.

## Supplementary information

Supplemental Table 1
