# Supplementary material for: Hepcidin mediates hypoferremia and reduces the growth potential of bacteria in the immediate post-natal period in human neonates
Source: Sci Rep. 2019 Nov 12;9:16596. doi: 10.1038/s41598-019-52908-w (PMC6851364; doi:10.1038/s41598-019-52908-w)
Supplement: Supplementary file 1 — Supplemental Table 1 [file 41598_2019_52908_MOESM1_ESM.docx]

Title: **Hepcidin mediates hypoferremia and reduces the growth potential of bacteria in the immediate post-natal period in human neonates**

**Authors:** Sarah Prentice ^1,2^ *, Amadou T. Jallow^2^, Edrissa Sinjanka^2^, Momodou W. Jallow^2^, Ebrima A. Sise^2^, Noah J. Kessler ^2,3^, Rita Wegmuller^2^, Carla Cerami^2^, Andrew M. Prentice ^2,3^

^1^ Clinical Research Department, London School of Hygiene and Tropical Medicine, Keppel Street, London, WC1E 7HT

^2^ MRC Unit The Gambia at London School of Hygiene & Tropical Medicine, Atlantic Road, Fajara, The Gambia and MRC Keneba.

^3^ Department of Genetics, University of Cambridge, Downing Street, CB2 3EH

^4^ Department of Health Sciences and Technology, ETH Zurich, Zurich, Switzerland

*Corresponding author.

**Supplementary Materials**

Table S1: Pearson's pairwise correlations of iron metabolism markers by post-natal age

|  | | Cord TSAT | Cord Iron | Cord TIBC | Cord Hepcidin | Cord IL6 | Cord Hb | <24hr  TSAT | <24hr  Iron | <24hr  TIBC | <24hr  Hepcidin | <24hr  IL6 | <24hr  Hb | 24-48hr  TSAT | <24-48hr  Iron | <24hrs–48hr  TIBC | <24hrs–48hr Hepcidin | <24hrs–48hr  IL6 | <24hrs-48hr  Hb | 72-96hr  TSAT | 72-96hr Iron | 72-96hr TIBC | 72-96hr Hepcidin | 72-96hr IL6 | 72-96hr Hb |
| --- | --- | --- | --- | --- | --- | --- | --- | --- | --- | --- | --- | --- | --- | --- | --- | --- | --- | --- | --- | --- | --- | --- | --- | --- | --- |
| Cord TSAT | **r**  **p** | 1.0 |  |  |  |  |  |  |  |  |  |  |  |  |  |  |  |  |  |  |  |  |  |  |  |
| Cord Iron | **r**  **p** | 0.81  0.0000 | 1.0 |  |  |  |  |  |  |  |  |  |  |  |  |  |  |  |  |  |  |  |  |  |  |
| Cord TIBC | **r**  **p** | -0.48  0.0000 | 0.04  0.70 | 1.0 |  |  |  |  |  |  |  |  |  |  |  |  |  |  |  |  |  |  |  |  |  |
| Cord Hepcidin | **r**  **p** | 0.02  0.86 | -0.37  0.0007 | -0.37  0.009 | 1.0 |  |  |  |  |  |  |  |  |  |  |  |  |  |  |  |  |  |  |  |  |
| Cord IL6 | **r**  **p** | -0.29  0.03 | 0.21  0.13 | 0.29  0.03 | -0.01  0.91 | 1.0 |  |  |  |  |  |  |  |  |  |  |  |  |  |  |  |  |  |  |  |
| Cord Hb | **r**  **p** | 0.01  0.92 | 0.11  0.37 | 0.24  0.06 | -0.10  0.41 | -0.05  0.75 | 1.0 |  |  |  |  |  |  |  |  |  |  |  |  |  |  |  |  |  |  |
| <24hr TSAT | **r**  **p** | 0.54  0.0001 | 0.55  0.0001 | 0.03  0.86 | -0.33  0.02 | -0.17  0.35 | 0.08  0.61 | 1.0 |  |  |  |  |  |  |  |  |  |  |  |  |  |  |  |  |  |
| <24hr Iron | **r**  **p** | 0.43  0.0009 | 0.55  0.0000 | 0.20  0.15 | -0.43  0.0006 | -0.15  0.35 | 0.13  0.37 | 0.94  0.0000 | 1.0 |  |  |  |  |  |  |  |  |  |  |  |  |  |  |  |  |
| <24hr  TIBC | **r**  **p** | -0.13  0.38 | 0.23  0.12 | 0.64  0.0000 | -0.54  0.0001 | -0.08 0.66 | 0.15  0.35 | -0.005  0.97 | 0.33  0.02 | 1.0 |  |  |  |  |  |  |  |  |  |  |  |  |  |  |  |
| <24hr  Hepcidin | **r**  **p** | 0.04  0.75 | -0.02  0.83 | -0.14  0.23 | 0.68  0.0000 | 0.32  0.01 | -0.23  0.07 | -0.47  0.0004 | -0.49  0.0000 | -0.40  0.003 | 1.0 |  |  |  |  |  |  |  |  |  |  |  |  |  |  |
| <24hr IL6 | **r**  **p** | -0.40  0.004 | -0.32  0.02 | 0.14  0.33 | 0.27  0.05 | 0.37  0.01 | 0.05  0.75 | -0.40  0.01 | -0.34  0.02 | -0.03  0.86 | 0.38  0.004 | 1.0 |  |  |  |  |  |  |  |  |  |  |  |  |  |
| <24hr Hb | **r**  **p** | 0.04  0.72 | 0.24  0.05 | 0.22  0.08 | -0.35  0.003 | 0.33  0.02 | 0.21  0.08 | 0.18  0.23 | 0.27  0.05 | 0.36  0.02 | -0.22  0.07 | -0.02  0.92 | 1.0 |  |  |  |  |  |  |  |  |  |  |  |  |
| 24-48hr TSAT | **r**  **p** | 0.54  0.01 | 0.70  0.0004 | 0.07  0.77 | -0.50  0.02 | -0.32  0.28 | 0.21  0.46 | 0.39  0.19 | 0.39  0.15 | 0.16  0.61 | -0.11  0.63 | -0.65  0.02 | 0.31  0.20 | 1.0 |  |  |  |  |  |  |  |  |  |  |  |
| 24-48hr Iron | **r**  **p** | 0.31  0.15 | 0.67  0.0004 | 0.24  0.27 | -0.58  0.004 | -0.31  0.26 | -0.10  0.71 | 0.41  0.14 | 0.65  0.004 | 0.50  0.07 | -0.28  0.19 | -0.74  0.001 | 0.34  0.12 | 0.86  0.0000 | 1.0 |  |  |  |  |  |  |  |  |  |  |
| 24-48hr TIBC | **r**  **p** | -0.48  0.03 | 0.03  0.91 | 0.50  0.02 | -0.32  0.15 | -0.24  0.40 | -0.33  0.21 | 0.17  0.56 | 0.58  0.02 | 0.88  0.0000 | -0.48  0.03 | -0.56  0.04 | 0.08  0.73 | -0.13  0.57 | 0.38  0.09 | 1.0 |  |  |  |  |  |  |  |  |  |
| 24-48hr Hepcidin | **r**  **p** | -0.04  0.84 | 0.006  0.98 | 0.11  0.56 | 0.40  0.03 | 0.32  0.20 | -0.37  0.10 | -0.35  0.20 | -0.22  0.37 | 0.22  0.42 | 0.49  0.007 | -0.15  0.49 | 0.03  0.90 | -0.35  0.13 | -0.19  0.40 | 0.14  0.56 | 1.0 |  |  |  |  |  |  |  |  |
| 24-48hr IL6 | **r**  **p** | -0.58  0.008 | -0.49  0.02 | 0.34  0.14 | -0.12  0.60 | 0.13  0.64 | 0.47  0.09 | -0.27  0.44 | -0.23  0.43 | -0.14  0.71 | 0.14  0.53 | 0.37  0.12 | 0.15  0.52 | -0.27  0.39 | -0.26  0.34 | 0.16  0.63 | 0.03  0.90 | 1.0 |  |  |  |  |  |  |  |
| 24-48hr Hb | **r**  **p** | 0.06  0.79 | 0.08  0.70 | 0.21  0.35 | -0.10  0.64 | 0.27  0.38 | 0.28  0.22 | 0.28  0.32 | 0.28  0.27 | 0.16  0.57 | -0.12  0.59 | -0.13  0.61 | 0.41  0.05 | 0.23  0.39 | 0.31  0.21 | 0.11  0.68 | 0.10  0.66 | 0.39  0.13 | 1.0 |  |  |  |  |  |  |
| 72-96hr TSAT | **r**  **p** | 0.36  0.04 | 0.79  0.0000 | -0.24  0.17 | -0.24  0.15 | 0.42  0.03 | -0.08  0.68 | 0.22  0.25 | 0.15  0.41 | -0.31  0.11 | 0.13  0.43 | 0.30  0.18 | 0.23  0.15 | na* | na | na | na | na | na | 1.0 |  |  |  |  |  |
| 72-96hr Iron | **r**  **p** | 0.05  0.78 | 0.10  0.58 | 0.05  0.81 | -0.22  0.21 | -0.32  0.16 | -0.07  0.70 | 0.18  0.38 | 0.21  0.27 | 0.02  0.93 | -0.12  0.48 | 0.25  0.28 | 0.18  0.34 | na | na | na | na | na | na | 0.87  0.0000 | 1.0 |  |  |  |  |
| 72-96hr TIBC | **r**  **p** | -0.40  0.03 | -0.18  0.34 | 0.50  0.005 | -0.37  0.03 | 0.63  0.004 | 0.24  0.22 | -0.11  0.59 | -0.05  0.80 | 0.16  0.44 | -0.21  0.24 | -0.27  0.28 | 0.22  0.25 | na | na | na | na | na | na | -0.30  0.09 | 0.04  0.81 | 1.0 |  |  |  |
| 72-96hr Hepcidin | **r**  **p** | -0.003  0.99 | -0.35  0.02 | -0.35  0.02 | 0.61  0.0000 | -0.27  0.12 | -0.23  0.13 | -0.35  0.05 | -0.32  0.05 | -0.13  0.47 | 0.32  0.02 | 0.21  0.26 | -0.35  0.01 | na | na | na | na | na | na | -0.16  0.33 | -0.10  0.59 | -0.46  0.009 | 1.0 |  |  |
| 72-96hr IL6 | **r**  **p** | -0.15  0.44 | -0.40  0.04 | 0.06  0.76 | -0.03  0.86 | -0.21  0.31 | -0.09  0.66 | 0.06  0.81 | 0.23  0.31 | 0.66  0.004 | -0.33  0.08 | 0.26  0.24 | -0.05  0.80 | na | na | na | na | na | na | -0.70  0.0001 | 0.02  0.92 | -0.45  0.04 | 0.15  0.41 | 1.0 |  |
| 72-96hr Hb | **r**  **p** | -0.06  0.75 | 0.11  0.54 | 0.31  0.08 | -0.62  0.0001 | -0.26  0.25 | 0.46  0.007 | 0.25  0.25 | 0.29  0.14 | 0.52  0.01 | -0.5  0.003 | -0.07  0.77 | 0.67  0.0000 | na | na | na | na | na | na | -0.16  0.43 | 0.13  0.51 | 0.36  0.06 | -0.27  0.13 | 0.17  0.48 | 1.0 |

* Due to limitations when blood-sampling from neonates, participants were randomly allocated to have a blood sample taken either at 24-48 hours of age or 72-96 hours of age. Therefore correlations between these two time points are not available. P-values <0.05 are highlighted in blue, p-values <0.01 are highlighted in red.
